# The first case of COVID-19 pneumonia in a hemodialysis patient in Japan

**DOI:** 10.1007/s13730-020-00495-5

**Published:** 2020-06-18

**Authors:** Yusuke Kuroki, Kazutoshi Hiyama, Junya Minami, Miyoshi Takeuchi, Masumi Shojima, Shumei Matsueda, Hiroshi Nagae, Toshiaki Nakano

**Affiliations:** 1 Division of Nephrology, National Hospital Organization Fukuokahigashi Medical Center, Koga, Japan; 2 Division of Infectious Disease, National Hospital Organization Fukuokahigashi Medical Center, Koga, Japan; 3 Department of Medicine and Clinical Science, Graduate School of Medical Sciences, Kyushu University, Fukuoka, Japan

**Keywords:** COVID-19, Hemodialysis, Hydroxychloroquine, Pneumonia, Fluid management, Dialysis membrane, Clot formation

## Abstract

On 31 December 2019, cases of pneumonia whose cause was later identified as SARS-CoV-2 were detected in Wuhan City, Hubei Province of China, and now COVID-19 has spread worldwide. On March 1, 2020, a 69-year-old Japanese man who had been on hemodialysis for 3 years was diagnosed as having COVID-19 pneumonia and hospitalized at our Medical Center. Pulmonary CT revealed bilateral multiple consolidation with bilateral pleural effusion. Aggressive weight reduction was needed to improve the patient’s respiratory condition. Hemodialysis therapy was performed in isolation with hydroxychloroquine administration, but the formation of a dialysis membrane clot forced the withdrawal of dialysis therapy. Changing the dialysis membrane material and anticoagulant enabled the resumption of dialysis therapy, allowing the body weight to correct downward. On the 5th hospitalization day, the patient’s fever dropped and he showed improved oxygenation and chest X-ray. He was eventually discharged. The hydroxychloroquine and appropriate fluid management may have contributed to the patient’s recovery. Clinicians should pay close attention to avoid dialysis-related problems when treating a patient with COVID-19.

## Introduction

On 31 December 2019, the World Health Organization (WHO) China Country Office was informed of cases of pneumonia of unknown etiology (unknown cause) detected in Wuhan City, Hubei Province of China [1]. A novel coronavirus was later identified as the cause of this outbreak: SARS-CoV-2 (severe acute respiratory syndrome coronavirus 2). The WHO named the disease caused by this novel coronavirus ‘COVID-19’. On March 11, 2020, the WHO declared a pandemic based on the worldwide spread of COVID-19. As of April 9, 2020, 212 countries, areas, and territories were confirmed to have COVID-19-infected individuals.

Dialysis therapy was provided for an estimated 2.6 million people worldwide in 2010, and this number is expected to increase [2]. If COVID-19 infection continues to spread, it is highly possible that the disease will spread to patients with end-stage kidney disease (ESKD). Here, we describe our experience with a first case of COVID-19 pneumonia in a maintenance hemodialysis patient in Japan. We provide the details of our patient’s case herein, focusing on the precautions needed when performing dialysis therapy for patients with COVID-19.

## Case presentation

A 69-year-old Japanese man was admitted to our hospital on March 1, 2020, with the complaint of fever, cough, and dyspnea. He had been diagnosed with diabetes 14 years earlier. Approximately 3 years before this admission, he had begun maintenance hemodialysis therapy because of his ESKD due to diabetic nephropathy. He was a smoker (~ 40 cigarettes/day). He was a taxi driver, and it was not clear whether any of his customers within the past 2 weeks had shown a fever or cough in his taxi. Because he was positive for an influenza test 16 days prior to his admission, oseltamivir phosphate was prescribed.

The patient’s fever was relieved 9 days prior to his admission with a negative result on a repeat influenza test, and he returned to work. However, fever was observed again 6 days prior to his admission. On chest X-ray, he was diagnosed with pneumonia, admitted to the hospital where he received maintenance hemodialysis therapy, and administered meropenem hydrate. However, his reaction to antibiotics was poor and his respiratory condition worsened. On the day of his transfer to our hospital, his pharyngeal swab sampled for SARS-CoV-2 was confirmed to be positive by a polymerase chain reaction (PCR) assay, and he was admitted to our hospital.

On admission, the patient was conscious and had a severe cough. His body temperature was 37.3 °C, and his blood pressure was 183/73 mmHg. Eight liters per min oxygen with a reservoir mask was necessary to maintain sufficient oxygen saturation. Endotracheal intubation was not performed, in accord with the patient’s wishes. Blood tests revealed an elevated white blood cell count (9.4 × 10^9^/L, normal 3.3–8.6 × 10^9^/L), elevated C-reactive protein (CRP) level (15.2 mg/dL on admission and 20.5 mg/dL at peak, normal < 0.3 mg/dL), and elevated brain natriuretic peptide (BNP) level (370 pg/ml, normal < 18.4 pg/ml). We suspected that the elevated BNP was due to fluid overload.

**Fig. 1:**
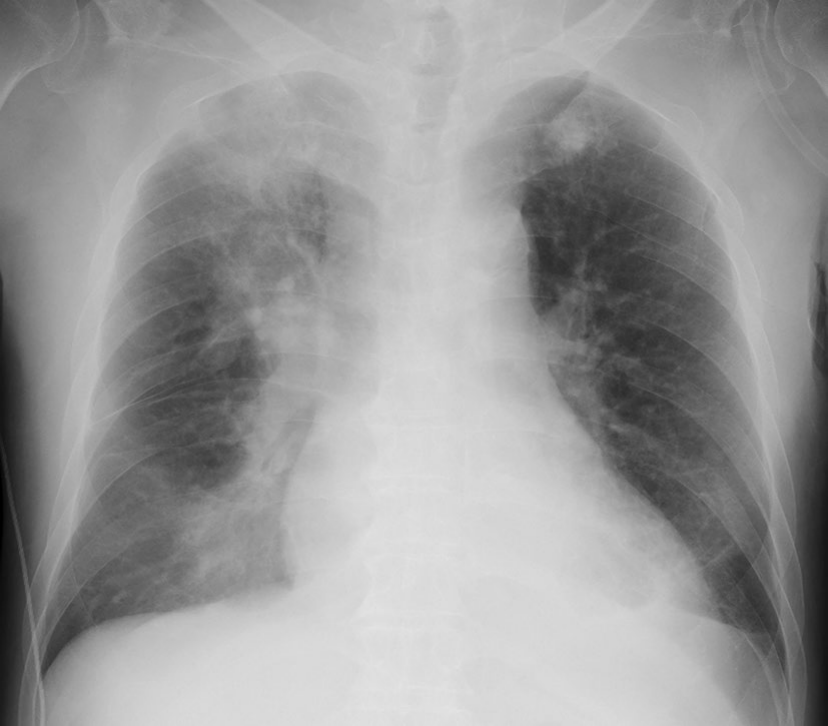
Chest X-ray on admission. Pulmonary consolidation on the right upper and lower lung of the patient, a 69-year-old Japanese male

**Fig. 2:**
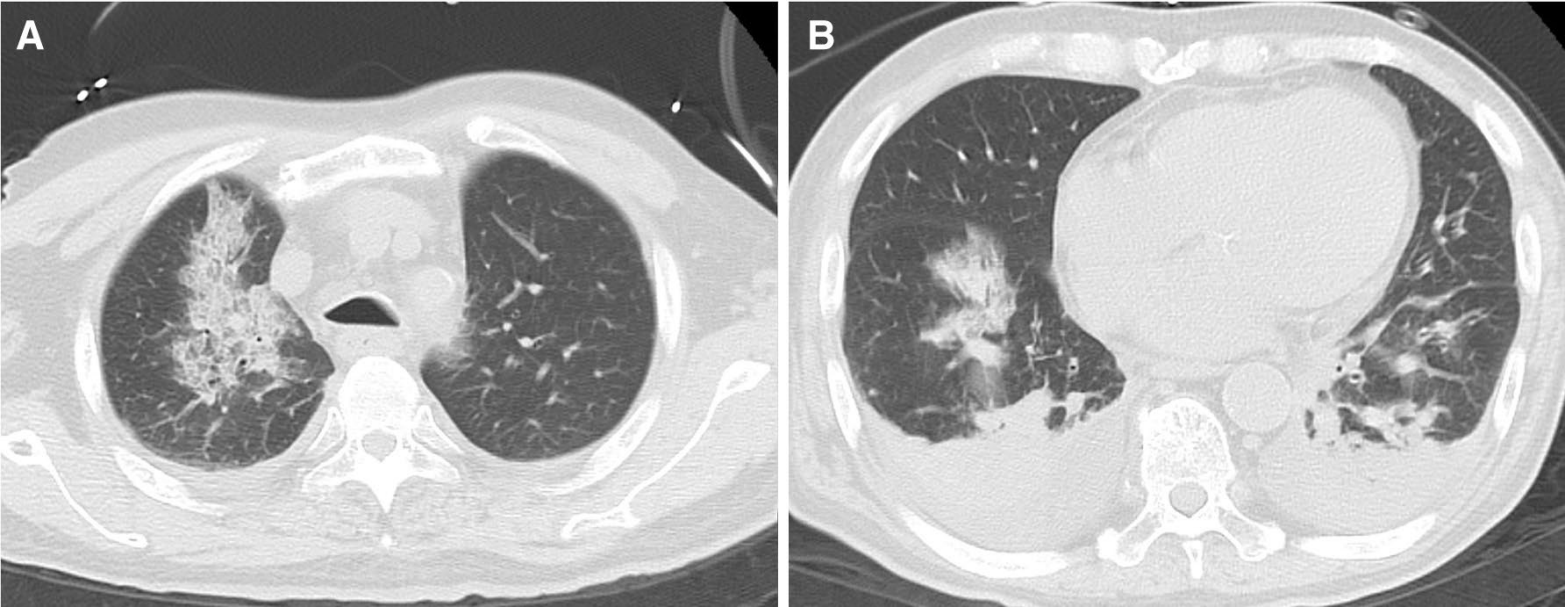
Pulmonary CT on the day before admission. **a**, **b** Bilateral multiple consolidation and ground-glass opacity. **b** Bilateral pleural effusion was seen

The chest X-ray on admission revealed pulmonary consolidation on the right upper and lower lung (Fig. 1). Pulmonary CT on the day before admission revealed bilateral multiple consolidation and ground-glass opacity. Bilateral pleural effusion was also confirmed (Fig. 2). An infectious disease specialist physician administered meropenem hydrate, levofloxacin hydrate, and the antiviral drug peramivir. Hydroxychloroquine (HCQ) 400 mg daily was administered for antiviral therapy. On the 2nd hospitalization day, hemodialysis therapy was performed for the patient under isolation conditions. Considering the need to reduce the patient’s pleural effusion, we planned an aggressive weight reduction for him.

Because the patient’s blood pressure before dialysis was stable at 162/78 mmHg, the hemodialysis therapy was started with a polysulfone membrane for the dialyzer, a bolus of unfractionated heparin (1000 units) for the initial dose, and 1000 units/h for the maintenance dose as an anticoagulant; these were the same hemodialysis conditions as before the patient’s admission. At approximately 3.5 h after the start of the hemodialysis, an elevation of transmembrane pressure (TMP) due to the formation of a dialysis membrane clot occurred. It became difficult to continue the hemodialysis, and the hemodialysis had to be discontinued. The antithrombin III (ATIII) level was within normal range.

**Fig. 3:**
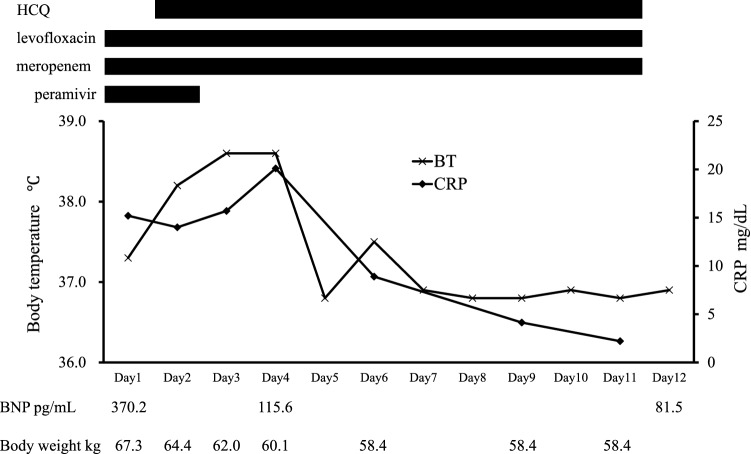
Drug use and changes in CRP levels, body temperatures, BNP levels and body weight. *HCQ* hydroxychloroquine, *BT* body temperature, *BNP* brain natriuretic peptide

On the 3rd hospitalization day, hemodialysis was performed again with an increase in the dosage of unfractionated heparin to 1500 units for the initial dose and 1500 units/h for the maintenance dose, but we had to stop the hemodialysis at 3 h after its initiation for the same reason as the day before. On the 4th hospitalization day, to avoid the clotting of the dialysis membrane, we changed the dialysis membrane to one made of polymethylmethacrylate (PMMA), and we changed the anticoagulant to nafamostat mesilate (50 mg/h for the maintenance dose). Thereby, the dialysis therapy could be continued with the downward correction of body weight after the dialysis. On the 5th hospitalization day, the patient’s fever had dropped with his improved oxygenation. On the 6th day, the oxygen administration was discontinued, and his body weight after dialysis reached 60.1 kg from 67.0 kg on admission with the body weight correction. A chest X-ray revealed improvement of the pulmonary consolidation on the right upper and lower lung, and a decreased CRP level (8.0 mg/dL) and BNP level (105 pg/dL) were observed; after that, the HCQ was discontinued (Fig. 3). On the 17th hospitalization day, the patient was repeatedly confirmed to be negative for SARS-CoV-2 by a PCR assay. He was discharged on the 19th day of hospitalization.

## Discussion

We identified COVID-19 pneumonia in a patient who had been undergoing maintenance hemodialysis therapy. After the administration of HCQ, adequate dialysis therapy, and a downward correction of the patient’s body weight, his respiratory condition improved and eventually his COVID-19 pneumonia was resolved [3].

Patients on dialysis therapy must be extra vigilant to avoid COVID-19 infection. For individuals who are on hemodialysis, infectious complications are a main cause of morbidity, mortality, and hospitalization [3]. Respiratory mortality is estimated to be 14–16 times higher in dialysis patients compared to the general population [4]. Infection seems to contribute to the cardiovascular disease burden in patients on dialysis [3]. It is highly probable that people undergoing hemodialysis could die from COVID-19 infection. The Japanese Association of Dialysis Physicians reported that as of May 8, 2020, 76 dialysis patients in Japan suffered from COVID-19 infection, and 9 had died [5]. The mortality rate was thus > 10%.

In Table 1, we have summarized 11 cases: the 8 reported cases, our present patient’s case, and the cases of 2 more hemodialysis patients with COVID-19 infection that we encountered after the present case [6–9]. The sex of one patient [6] was not reported; seven of the other ten patients were male. Fever was observed in 7 and diarrhea was observed in 5 of the 11 patients. Eight of the nine patients whose lymphocyte counts were reported had lymphopenia, defined as < 1000 × 10^6^/L. The rate of lymphopenia was higher than that of fever.

**Table 1:** Summary of COVID-19 infection in hemodialysis patients

References	Age	Sex	Dialysis vintage, years	Cause of ESKD	Fever	Dyspnea	Diarrhea	WBC count, × 10^6^/L	Lymphocyte count, × 10^6^/L	Antiviral medications	Outcome
Tang et al. [6]	50 s	Unknown	Unknown	DN	No	No	No	3380	Unknown	Lopinavir/ritonavir	Recovered
Fu et al. [7]	75	Male	Unknown	Kidney atrophy	No	Unknown	No	7200	280	Oseltamivir	Recovered
Ferrey et al. [8]	56	Male	3	IgAN	Yes	Yes	Yes	Unknown	Unknown	HCQ	Unknown
Wang et al. [9]	61	Male	7	HN	Yes	No	Yes	6840	630	1 Arbidol 1 Rivabirin	Unknown
	62	Male	3	HN	No	No	No	7500	840		Unknown
	47	Female	5	HN	Yes	Yes	Yes	7730	800		Unknown
	67	Female	1	CN	Yes	Yes	Yes	10,760	920		Unknown
	51	Male	1	HT	No	No	Yes	5030	490		Unknown
Present case	69	Male	3	DN	Yes	Yes	No	9400	1010	HCQ	Recovered
Our 2nd case	25	Male	4*	CIN	Yes	No	No	2500	450	HCQ	Recovered
Our 3rd case	72	Female	6	DN	Yes	Yes	No	2800	430	HCQ	Recovered

*CIN* chronic interstitial nephritis, *CN* chronic nephritis, *DN* diabetic nephropathy, *ESKD* end-stage kidney disease, *HCQ* hydroxychloroquine, *HN* hypertensive nephropathy, *IgAN* IgA nephropathy

*He received a living donor-related renal transplantation because of ESKD 6 years earlier, but started hemodialysis because of graft loss 4 years ago

We administered HCQ as a therapeutic agent for the present patient’s COVID-19 pneumonia. When the patient was first hospitalized, there was little information about the effectiveness of antiviral drugs, and favipiravir and remdesivir were not available at our hospital. We selected HCQ as antiviral drug that has been reported to be effective for COVID-19 infection [10, 11]. Regarding HCQ, because the mean amount of HCQ excreted unchanged in the urine was 27% of the infusion dose [12], the blood levels may increase in patients with ESKD. In the present case, the usual amount of HCQ was administered as a short-term treatment, and it could be used without major adverse effects. Future studies are needed to determine the efficacy of HCQ in hemodialysis patients with COVID-19 pneumonia.

In hemodialysis patients, an inappropriate dry weight setting causes an elevated cardiac capacity load and the appearance of pleural effusion, resulting in the worsening of the patients’ respiratory condition. An elevated cardiac capacity load is associated with an increased risk of lung infection [13]. N-terminal pro-brain natriuretic peptide (NT-proBNP) is a predictive factor of pneumonia in hemodialysis patients [14]. In our patient’s case, we actively reduced his dry weight because of the bilateral pleural effusion and BNP elevation at the time of admission. This may have been involved in the improvement of his respiratory condition and the healing of his pneumonia.

In the patient’s first two dialysis sessions, unfractionated heparin could not prevent the formation of a dialysis membrane clot during the hemodialysis, requiring the withdrawal of hemodialysis despite the good control achieved with unfractionated heparin before the patient’s admission. In this case, no decrease in ATIII was observed, and the hemodialysis could be continued by changing the anticoagulant and the dialysis membrane. Intracircuit clot formation during extracorporeal circulation was reported to be associated with high levels of polymorphonuclear granulocyte (PMN) elastase, which activates blood coagulation and inactivates ATIII, derived from leukocytes in contact with the dialysis membrane [15]. It was reported that PMNs from ESKD patients expressed tissue factor (TF; the major trigger of coagulation in vivo) during the course of hemodialysis sessions in response to complement anaphylatoxin C5a [16]. It has also been reported that COVID-19 infection was associated with thrombosis [17]. The thrombogenicity in our patient’s case may have been associated with his COVID-19 infection.

It is desirable to reduce the frequency of alarm ringing under isolated hemodialysis conditions to avoid secondary infections to health-care workers and to prevent intracircuit clotting because of extracorporeal circulation stoppage. Considering the possibility of dialysis membrane clotting, it may be necessary to take prompt safety measures for hemodialysis patients who have developed COVID-19 pneumonia, such as changing the material of the dialysis membrane or changing the anticoagulant, as in our patient’s case. It was reported that a dialysis patient with COVID-19 infection was treated successfully with continuous renal replacement therapy (CRRT) [7]. Although CRRT is a useful treatment option, in our patient’s case CRRT may not have been advisable, due to the thrombogenicity of the dialysis membrane and difficulty in maintaining sufficient rest. Clinicians should pay close attention to circuit coagulation when considering the application of CRRT.

In conclusion, we successfully treated a patient with diabetes and COVID-19 pneumonia who was undergoing maintenance hemodialysis therapy. The HCQ administration and appropriate fluid management may have contributed to the patient’s recovery. Clinicians should pay close attention to avoid dialysis-related problems such as dialysis membrane clot formation.
